# The Inhibition of the Rayleigh-Taylor Instability by Rotation

**DOI:** 10.1038/srep11706

**Published:** 2015-07-01

**Authors:** Kyle A. Baldwin, Matthew M. Scase, Richard J. A. Hill

**Affiliations:** 1 School of Physics and Astronomy, University of Nottingham, Nottingham, NG7 2RD, UK; 2 School of Mathematical Sciences, University of Nottingham, Nottingham, NG7 2RD, UK

## Abstract

It is well-established that the Coriolis force that acts on fluid in a rotating system can act to stabilise otherwise unstable flows. Chandrasekhar considered theoretically the effect of the Coriolis force on the Rayleigh-Taylor instability, which occurs at the interface between a dense fluid lying on top of a lighter fluid under gravity, concluding that rotation alone could not stabilise this system indefinitely. Recent numerical work suggests that rotation may, nevertheless, slow the growth of the instability. Experimental verification of these results using standard techniques is problematic, owing to the practical difficulty in establishing the initial conditions. Here, we present a new experimental technique for studying the Rayleigh-Taylor instability under rotation that side-steps the problems encountered with standard techniques by using a strong magnetic field to destabilize an otherwise stable system. We find that rotation about an axis normal to the interface acts to retard the growth rate of the instability and stabilise long wavelength modes; the scale of the observed structures decreases with increasing rotation rate, asymptoting to a minimum wavelength controlled by viscosity. We present a critical rotation rate, dependent on Atwood number and the aspect ratio of the system, for stabilising the most unstable mode.

The Rayleigh-Taylor instability (RTI) is a fundamental fluid dynamical phenomenon that occurs at the interface between a dense fluid supported by a lighter fluid under gravity, or, equivalently, in a system that is accelerating in the direction of the denser fluid. An example is the instability of a layer of brine above a layer of fresh water. The RTI has remained topical since the initial work of Lord Rayleigh^1^ and the investigations of Taylor^2^ and Lewis^3^. Reasons for this include its common occurrence in industrial applications involving, for example, thin film flows^4^,^5^, pool boiling^6^ and aerosol transport^7^, as well as its intrinsic mathematical value^8^,^9^. It manifests in several natural physical phenomena including, for example, the finger-like structures of the Crab nebula, created by the acceleration of pulsar winds through a dense supernova remnant^10^, and salt tectonics, where deep salt sediments protrude upwards through denser rock into finger-like “diapirs”^11^. The RTI also constrains efforts to generate power from inertial confinement fusion, where, during the implosion of the target, instabilities develop at the interface between the vaporized shell and the fuel core, limiting the efficiency of the reaction^12^,^13^,^14^,^15^,^16^,^17^. These examples illustrate the importance of furthering our understanding and our ability to influence the RTI through means beyond, for example, imposing an initial density difference. In particular there are many situations in which it is desirable to stabilise the system against RTI, as in inertial confinement fusion.

Rotation can have a stabilising effect on an inherently unstable flow (see *e.g.* Fultz^18^ and references therein) by inhibiting velocity gradients parallel to the axis of rotation. This tends to impede large scale overturning at an interface perpendicular to the axis of rotation. Based on numerical simulations, Carnevale *et al.* assert that rotation can stabilise indefinitely the RTI in a regime where mass diffusion is comparable to momentum diffusion^19^, *i.e.* for gas phase flow in which the Schmidt number Sc ~ *O*(1). More recently, Tao *et al.*^20^ also considered, theoretically, using rotation to inhibit the RTI in the case where the rotation axis is *perpendicular* to the direction of acceleration, a situation akin to a fluid-fluid centrifuge. Here we show experimentally that rotation of a liquid system (Sc ~ 10^4^) about an axis *parallel* to the direction of acceleration slows the growth of the instability and changes its character in a rigidly rotating three-dimensional liquid system (Fig. 1).

In studying the RTI experimentally, a difficulty arises in maintaining the stability of the heavy fluid above the lighter fluid during preparation of the system. One method for overcoming this is to use rocketry^21^ or other means of acceleration (*e.g.* compressed gas^2^,^3^,^22^, linear electric motors^23^) to induce RTI in a gravitationally-stable two-fluid system by suddenly inverting the direction of acceleration. This method fails when the two fluid system is rotated, for the following reasons. The interface forms a paraboloid as a result of the centripetal acceleration required to keep the liquid in solid body rotation. For example, a vigorously stirred drink has a concave parabolic free interface with its lowest point on the axis of rotation. The isobaric surfaces form identically-shaped ‘concave’ parabolas, independent of density and density gradient. If the direction of acceleration is suddenly inverted, at the moment this occurs, the shape of the isobaric surfaces is also inverted, becoming ‘convex’. However, it takes a finite time for the shape of the interface to follow. Since the interface does not coincide with an isobar at the moment of inversion, the RTI does not develop from hydrostatic conditions. Another method for creating unstable initial conditions is to employ a barrier between the two fluids during preparation, but the same curvature of the interface presents significant technical difficulties for this technique. These barrier removal methods also perturb the fluid interface at the instant the barrier is withdrawn due to viscous drag and liquid displacement^24^, and the wake induced by the removal of the barrier may dominate the initial growth of the instability^25^.

Recently, magnetic fields have been used to induce RTI constrained to two dimensions^26^,^27^ and rotating magnetic fields used to stabilise a cylindrical ferrofluid system^28^. The approach we adopt here is to use the magnetic field of a superconducting magnet (Cryogenic Ltd. London) to manipulate the *effective* density of the two liquids in order to induce RTI in a fully three-dimensional system. Previously, this technique has been applied to float dense objects in less dense fluids (see *e.g.*^29^,^30^,^31^), exploited as a method for separating granular materials^32^, and used to influence thermal convection^33^.

Here, a light, paramagnetic liquid was floated upon a denser diamagnetic liquid layer in a transparent cylindrical acrylic tank approximately 10 cm in diameter. The top and bottom layers consisted of an aqueous solution of manganese chloride and an aqueous solution of sodium chloride respectively. The Methods section contains further details. The tank was placed on a rotating platform above the magnet and rotated until the liquids were rotating as a rigid body, before being allowed to descend at a constant vertical speed into the magnetic field, with no change in angular velocity. Figure 2 shows a schematic diagram of the experimental set-up.

## Results

As the tank descended into the magnetic field, the downward magnetic force on the upper liquid and the upward magnetic force on the lower liquid increased. This magnetic force is a body force: the force per unit volume is given by **F** = *χB*∇*B*/*μ*_0_, where *B* is the magnitude of the magnetic field, *μ*_0_ is the magnetic constant, and *χ* is the magnetic susceptibility, which is positive for the paramagnetic liquid (upper layer) and negative for the diamagnetic liquid (lower layer). Directly above the magnet bore the direction of ∇*B*, and therefore the magnetic force, lies close to vertical, so that the gravitational and magnetic body forces may be subsumed into a single, vertically-acting ‘effective gravitational’ force. Equivalently, we can consider that the force results from a change in the effective density ρ^*^ of the liquid in the magnetic field. In this picture the net vertical body force per unit volume is given by *F*_*z*_ = −ρ^*^g, where 
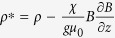
, and the effective Atwood number is then simply defined as 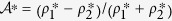, where 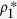 is the effective density of the upper fluid layer, and 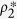 is the effective density of the lower fluid layer. In manipulating the relative magnitudes of the effective densities, the equilibrium profile is unaffected, and the RTI is initiated from hydrostatic conditions.

In Fig. 3, contours (blue lines) show the effective Atwood number, 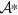, versus vertical and radial distance from the centre of the magnet. Note that 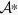 depends on position since ρ^*^ depends on the strength of the magnetic field and its vertical gradient, both of which depend on position. The red dashed lines show the curvature of the hydrostatic parabolic interface for rotation rates of Ω = *π*, 2*π*, 3*π* and 4*π* rad s^−1^, where the interface crosses 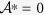. The grey dotted lines denote magnetic field lines.

### Rate of growth

**Figure 1:**
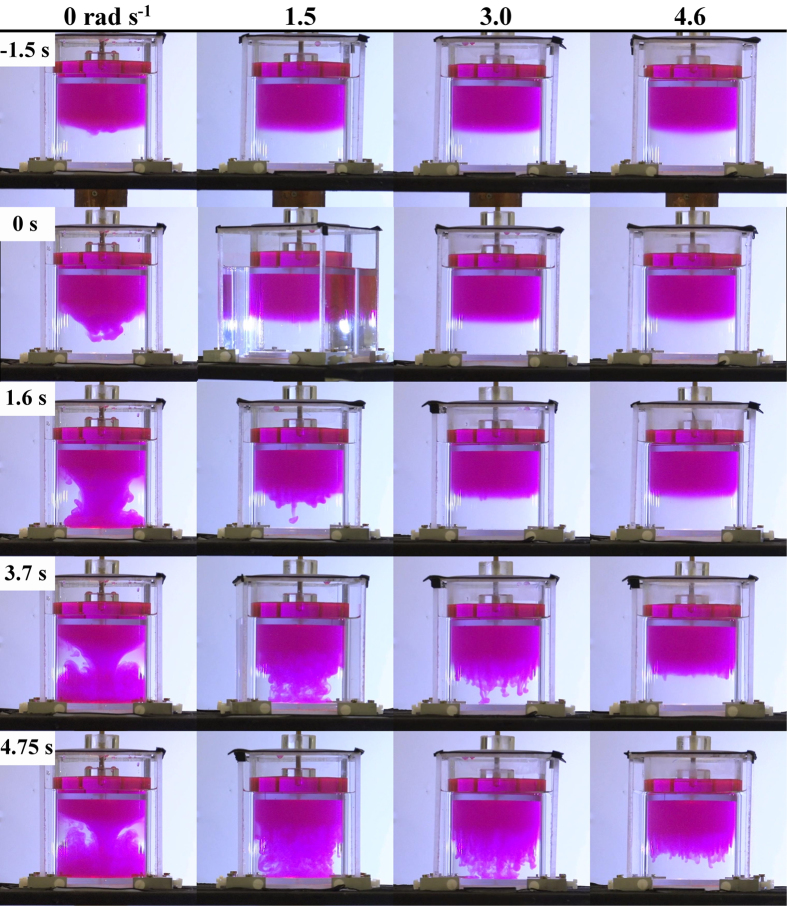
Rotation slows the growth of the RTI: each image shows a transparent cylindrical tank (inside a transparent box 12 × 12 × 14 cm) containing a paramagnetic liquid (pink) above a diamagnetic liquid (colourless). Each column of images shows the growth of the instability for a particular rotation rate as the tank is lowered into the magnetic field. A video, which is the source of the images shown in the figure, is included in the Supplementary Information on-line.

The descent of the rotating platform was halted when 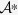 at the interface reached a small positive value, 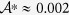, whereupon the interface became unstable, developing undulations that grew into characteristic finger-like structures. Figure 1 shows images of the interface viewed from the side for varying rotation rates and various times after the onset of instability. A video, which is the source of the images shown in the figure, is included in the Supplementary Information on-line. In the non-rotating experiment, the onset of RTI is apparent prior to the tank coming to a halt at *t* = 0 s. The images show that the early growth of the instability is slowed significantly with increasing rotation rate.

In order to obtain a quantitative measure of this suppression, the vertical extent of the growing ‘fingers’ of fluid was measured as a function of time. We created a ‘time series image’ in which each video frame is represented by a single column of pixels, such that the horizontal axis of the resultant image represents elapsed time; a typical example is shown in the inset to Fig.4. The growth of the fingers is visible in such images as a pink column of pixels that descends from the position of the interface, with time increasing from left to right. The Supplementary Information contains details of the algorithm used to produce these images. We identified a time *t* = *T* at which the scale of the fingers exceeded an arbitrary threshold, in this case 2 mm, *i.e. T* is defined by *h*(*T*) = −2 mm. The error in the measurements reflects noise in the images. Figure 4 shows that *T* increases up to a rotation rate of Ω ≈ 6 rad s^−1^, beyond which the threshold time does not increase, within experimental uncertainty.

### Wavelength of the most rapidly growing mode

We now consider how rotation affects the character of the instability, for various Ω and times after onset of RTI. In these experiments, the interface was filmed from above the tank, and the platform was allowed to descend during the entire course of RTI mixing. Figure 5a shows still images from the video, at rotation rates Ω between 0 and 13.2 rad s^−1^ and for times up to 2 s after the onset of the instability. An example video is included in the Supplementary Information, on-line. At the onset of RTI, undulations appeared at the interface, which grew in amplitude with time. The character of the undulations changed with increasing Ω: for small Ω, the disturbance grew as a cellular structure, but with increasing Ω this was replaced by more meandering snake-like concentric structures. These ‘snakes’ had a dominant radial wavelength and dominant azimuthal wavelength. The dominant radial wavelength was relatively insensitive to time, whereas the azimuthal wavelength decreased with time after onset of RTI, approaching that of the radial wavelength, whereupon the concentric patterns broke up into finger-like structures. Most noticeably, the radial wavelength decreased with increasing Ω.

To characterize the dominant radial wavelength of the instability, *λ*, we computed the two-dimensional auto-correlation function of the image, 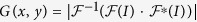, where *I*(*x*,*y*) is the grey-scale image in the interrogation window, and 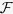 is the two-dimensional discrete Fast Fourier Transform. From *G*(*x*,*y*) we derived a one-dimensional autocorrelation function, 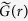, by averaging over azimuthal angle *θ*. A representative set of 1D autocorrelation traces for a given experiment (Ω = 5.15 rad s^−1^) is shown in Fig. 6. This example shows that the first peak appears at approximately 6 to 7 mm. We restricted our analysis to time less than 2 seconds after onset of RTI. The total time between onset of the RTI and the fingers reaching the lid and base of the tank was approximately 3 s.

Figure 7 shows a plot of *λ* as a function of Ω. The bars on each data point indicate the minimum and maximum value of *λ* measured over the first 2 seconds after onset of RTI. The plot shows that rotation caused a decrease in *λ* from 16 ± 2 mm at 1 rad s^−1^, to approximately 6 mm at 4 rad s^−1^, which is readily observable in the video images (see Fig. 5a). Below 1 rad s^−1^, the length scale was too large to resolve with the autocorrelation function. Above 4 rad s^−1^, increasing rotation rate had little effect on *λ*.

### Effect of viscosity

It is known that fluid viscosity suppresses small scale structures in the non-rotating RTI (*e.g.*^9^,^34^), suggesting that the observed lower limit (≈6 mm in Fig. 7) on the wavelength of the dominant mode may be dependent on the viscosity of the liquid. To verify this experimentally, equal quantities of glycerol were added to each layer. Figure 5b shows images of the interface for fixed rotation rate (Ω = 7.8 ± 0.1 rad s^−1^), and increasing viscosity, from top left to bottom right, at 1.6 s after onset. Figure 7 (inset) is a plot of *λ* versus viscosity, which shows a general trend from short to long wavelength instability as viscosity is increased.

## Discussion

In general, applying a magnetic field to a conducting liquid introduces magnetohydrodynamic effects. Here, however, magnetohydrodynamic forces are usually weak compared with the other body forces imposed on the system. The magnetic field affects the liquid primarily through its interaction with the spins of the electrons of the Mn^2+^ ions, and with the orbital motion of the electrons in the water molecules, rather than with macroscopic electric currents: the magnetic body force that destabilises the system is due to the magnetization of the liquid by the alignment of the spins of Mn^2+^ and by the orbital angular momentum imparted to the electrons by the imposition of the magnetic field^35^. Although magnetised, the magnetic field generated by magnetization of the liquid is insignificant compared to the imposed magnetic field, since the susceptibility of the liquids is of order 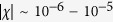 (unlike a ferrofluid for example). The Lorentz force **J** × **B** (per unit volume), responsible for magnetohydrodynamic effects, acts on ions carrying a current **J** in the liquid. Here, the current is given by **J** = *σ*(**u** × **B**) where **u** is the velocity of the liquid and *σ* is its conductivity. Note that, under rigid body rotation at a constant angular velocity, the Lorentz force does not influence the motion since there cannot be a purely radial electric current; the Lorentz force in this case is balanced by a radial electric field. Hence we need only consider the component of the Lorentz force that results from deviations in the velocity field from rigid body rotation. We can also assume that the magnetic field produced by any electric currents generated in the liquid is insignificant compared to the externally imposed field, since the conductivity of the liquid is relatively low *σ* ≈ 4 S m^−1^; the magnetic Reynolds number Re_m_ = *μ*_0_*ULσ* ~ 10^−7^ − 10^−8^ for the largest length (*L*) and velocity (*U*) scales considered here. To understand the effect that this force may have on the experiment, we consider the relative magnitudes of the Lorentz force, Coriolis force and viscous forces, all of which are proportional to the velocity of liquid motion deviating from rigid body rotation. The Elsasser number El = *σB*^2^/(*ρ*Ω) is a measure of the ratio of the Lorentz force to the Coriolis force; in these experiments, El was less than 2 × 10^−2^ for Ω ≥ 0.5 rad s^−1^, which is the smallest non-zero rotation applied experimentally, and less than 1 × 10^−2^ for Ω > 1 rad s^−1^. This implies that the effect of the Lorentz force is weak compared to the Coriolis force in these experiments. To verify this experimentally, we repeated the experiments, replacing the sodium chloride solution in the lower layer with a density-matched (hyphenated) zinc sulphate solution. We measured the conductivity of the zinc sulphate solution to be approximately 35% less than that of the sodium chloride solution of the same density. We found that the change in the growth rate with increasing rotation was the same in both cases, within experimental uncertainty, thus showing that the stabilising effect of rotation is unrelated to the motion of the liquid through a magnetic field (see Supplementary Information). We now consider the relative magnitude of the Lorentz and viscous forces, which is quantified by the Hartmann number, Ha^2^ = *B*^2^*L*^2^*σ*/(*ρν*). In experiments in which we did not add glycerol to the liquids, Ha^2^ exceeds 1 for length scales 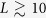 mm, indicating that, at these length scales, the Lorentz force has more influence on the motion of the liquid than viscosity. (Note that, even in this case, the magnitude of the Coriolis force in the rotating experiments is more than 50 times larger than that of the Lorentz force). Consistent with this, our experiments to determine the influence of viscosity on the observed lower limit of the dominant wavelength at a fixed rotation rate (inset Fig. 7), suggest a contribution from the Lorentz force to the effective viscosity of the liquid: extrapolation suggests that *λ* ≈ 5 mm even at *η* = 0. This viscous-like damping of a conducting fluid by Lorentz force is well-understood (see, for example^36^). Here, the Lorentz force appears to act to weakly dampen motion deviating from rigid-body rotation, although the damping effect is much weaker than that observed in a liquid metal for example, which has a much larger conductivity. The Supplementary Information contains plots of the Elsasser number, the Hartmann number and the Ekman number Ek = El/Ha^2^ as a function of Ω.

Owing to the spatial variation of *B*, the interface tends to become unstable at the centre of the tank slightly before the edges of the tank. This effect is enhanced for higher rotation rates owing to the parabolic curvature of the interface. For this reason we limited experiments to 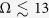 rad s^−1^. Ideally, the magnetic field should be imposed on the liquid instantaneously; however, due to the large inductance of the superconducting magnet, the rate at which we can increase *B* is too slow to induce RTI. Our approach of lowering the tank into the magnetic field achieves the objective of imposing the field quickly enough to induce RTI in the bulk of the system.

We now propose a mechanism through which rotation can act to stabilise the interface. With increasing rotation rate, a homogeneous liquid system tends to arrange itself into Taylor columns, parallel to the axis of rotation^37^. In a two layer system, the coherence of the columns is affected by the strength of the (effective) density difference at the interface. That is to say, the propensity for liquid to remain as a column competes with the RTI, in which liquid parcels in the upper and lower layers must move laterally relative to one another in order to overturn and rearrange themselves into a more stable configuration. The consequence of this competition is that relative lateral movement of fluid parcels either side of the interface is inhibited by virtue of the Taylor-Proudman Theorem^38^,^39^,^40^. As a result, with increasing rotation rate, liquid parcels switch position by forming smaller structures that require less lateral displacement to move past one another, and into a more stable arrangement. This results in the stabilisation of large wave modes that otherwise develop in the non-rotating case and hence we observe the development of shorter wavelength modes, decreasing in scale with increasing rotation rate. Since rotation appears to suppress wavelengths above a certain critical length scale, it might be anticipated that, for an inviscid system, as the rate of rotation increases the observed length scale of perturbation should tend toward zero. However, in our system, which has viscosity, we observe that the length scale asymptotes to a finite value that can be controlled by viscosity (≈ 6 mm in our set-up, see Fig. 7).

We can formalize the above argument by considering a generalization of the approach of Taylor^2^ to include rotation. Miles^41^ noted the importance of including the effects of the parabolic free-surface of a rotating body of liquid when calculating the properties of free-surface waves in rotating systems. Therefore, we combine the approaches of both Taylor^2^ and Miles^41^ and consider a two-layer liquid stratification in a cylindrical tank that is rotating about its axis with angular frequency Ω. The position of the initial interface is given by 
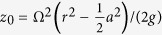
, relative to cylindrical coordinates whose origin coincides with the centre of the tank, and where *a* is the radius of the tank. Following the method outlined in Miles^41^, it can be shown that for low rotation rates, 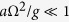, an axisymmetric instability at the interface develops as *e*^*iωt*^, where *t* is time and
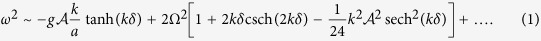

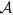
 is the Atwood number, *δ* is the aspect ratio of the cylinder (layer depth to radius) and *k* is a zero of the Bessel function *J*_1_, such that *k*/*a* is approximately the wavenumber associated with the mode of instability (cf. Miles^41^, (4.10)). For *ω*^2^ < 0 the mode is Rayleigh-Taylor unstable and grows. Equation (1) shows that, at least for low rotation rates, the growth rate of an unstable mode may be reduced by rotating the system. The second term on the right hand side of (1) may increase the value of *ω*^2^ and hence slow the growth rate. It can be further shown, without recourse to small rotation rate asymptotics, that an axisymmetric mode may be completely stabilised provided the critical rotation rate, Ω_*c*_, given by
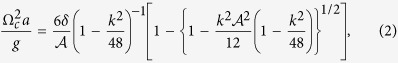
can be achieved without the interface intersecting the lid or base of the tank.

Considering the largest, mode 1, unstable mode, we may substitute our experimental parameters into (2) (*g* = 9.81 m s^−2^, *k* = 3.83, *δ* = 0.87, *a* = 4.50 × 10^−2^ m, 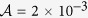) to find a critical rotation rate Ω_*c*_ = 1.18 rad s^−1^. This is consistent with the images in Fig. 1 where the dominant mode 1 instability, that spans the breadth of the tank in column 1 (Ω = 0 rad s^−1^ < Ω_*c*_), has been apparently stabilised in column 2 (Ω = 1.5 rad s^−1^ > Ω_*c*_).

Tao *et al.*^20^ recently speculated on the use of rotation to suppress RTI in the spherical pellets used in inertial confinement fusion. They concluded that rotating a pellet might retard the RTI during the acceleration phase, at the equator of the pellet. Our experiments suggest that the RTI at the poles of a rotating spherical fluid may also be suppressed. The present experiments are limited to A ~ *O*(10^–3^), but our theory shows that this result is applicable to any Atwood number. This implies that rotation could inhibit the growth of the instability over the entire surface of a rotating sphere.

Chandrasekhar considered the special case of infinite bodies of inviscid fluid separated by a horizontal interface, and concluded that an unstable configuration could not be stabilised indefinitely by rotation^9^. This analysis was further developed by Hide^42^ who included the effects of viscosity, and considered the wavenumber and growth rate of the most unstable modes. Our results agree qualitatively with Hide’s, who concluded that rotation slows the growth of the instability. However, quantitative comparison of our experimental results with Hide’s is problematic, since Hide’s analysis considers the growth of the most unstable modes at very short times after the onset of the instability. In comparison, the length scales we observe in our experiments are not necessarily those of the fastest growing initial mode of disturbance, as larger scale wave modes may overtake smaller scale wave modes before their presence is experimentally observable.

In conclusion, we have developed a method for magnetically inducing the Rayleigh-Taylor instability in a fully three-dimensional system, and have analysed the effect rotation has on the growth rate and the length scale of the instability. We found that rotation acts to suppress the growth of large wavelength undulations, with the wavelength of the fastest growing mode decreasing with increasing rotation rate, asymptoting to a fixed value that depends on the viscosity of the liquid. These data suggest that rotation acts to stabilise long wavelength instabilities, while viscosity stabilises short wavelength instabilities, producing an RTI whose character and growth are determined by the combined effects of both.

## Methods

Two liquid layers, consisting of an aqueous paramagnetic solution lying on top of an aqueous diamagnetic solution, were prepared in a cylindrical acrylic tank 10.7 cm in diameter. The paramagnetic solution was floated slowly onto the diamagnetic one to leave a well defined interface between the two. The top layer of liquid was a solution of manganese chloride (MnCl_2(aq)_, 0.06 mol l^−1^), and the bottom layer was a solution of sodium chloride (NaCl_(aq)_, 0.43 mol l^−1^). A transparent lid was submerged into the top layer such that there was no interface with the atmosphere and the two layer depths were equal. The volume magnetic susceptibilities and densities of the liquids were *χ*_1_ = 3.37 × 10^−6^ (SI units) and *ρ*_1_ = 998.2 ± 0.5 kg m^−3^ respectively for the top layer, and *χ*_2_ = −9.03 × 10^−6^ (SI units), *ρ*_2_ = 1012.9 ± 1.2 kg m^−3^ respectively for the bottom layer. The magnetic susceptibility was determined by means of a Gouy balance. The conductivity was *σ* ≈ 3 – 4 S m^−1^ in the lower layer, and approximately half that in the upper layer. The magnetic field strength was B ~ 1 − 2T. The fluid viscosity was varied for some experimental runs and lay in the range *ν* = *η*/*ρ* = 1 × 10^−6^ to 30 ×10^−6^ m^2^s^−1^depending on the concentration of glycerol used. The temperature of the tank and its contents was *T* = 22 ± 2 °C.

The rotation of the platform was achieved by means of a drive shaft connected to an electric motor. The rate of rotation was increased slowly, at approximately 0.002 rad s^−2^, up to the desired rate of rotation. The tank was left to rotate at this rate until the liquids were rotating as a rigid body; this was verified in separate experiments using neutrally buoyant tracer particles. A catch was then released allowing the platform to descend into the magnetic field. The tank was driven to rotate at the same speed during descent by means of a slip bearing. No longer than 2 hours elapsed between filling the tank and releasing the catch. Magnetic damping caused by eddy currents generated in the copper support cylinder kept the rate of descent constant over the duration of each experiment.

**Figure 2:**
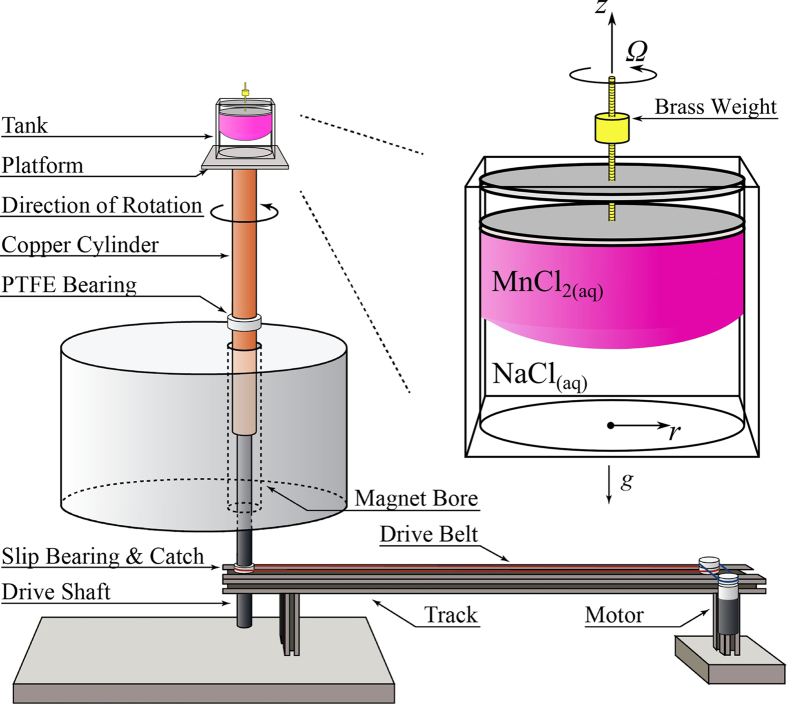
Schematic diagram of the experimental set-up. The cylindrical liquid tank, shown top right, is placed on the platform, spun up to a desired rotation rate (Ω), and lowered into the magnetic field at constant rate of descent while continuing to rotate. *r* and *z* are cylindrical coordinate axes.

**Figure 3:**
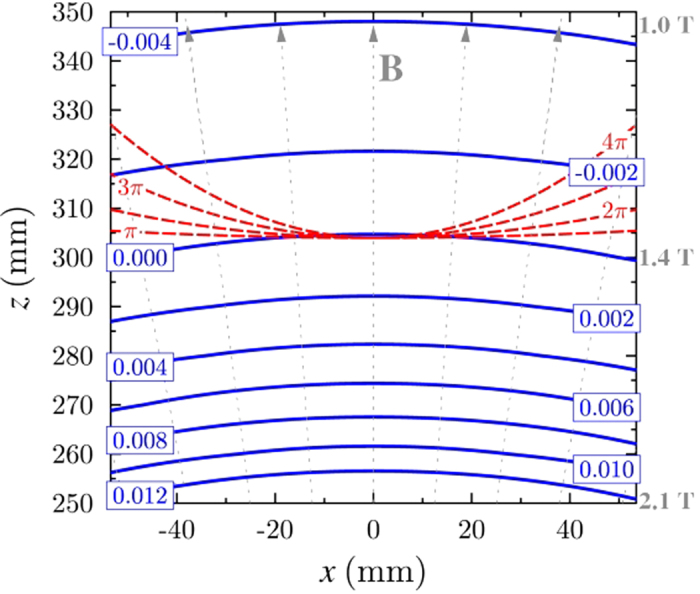
Blue contour lines show the spatial variation of the effective Atwood number. The origin *x* = 0, *z* = 0 is the geometric centre of the solenoid. The red dashed lines show the curvature of the hydrostatic parabolic interface for rotation rates of Ω = *π*, 2*π*, 3*π* and 4*π* rad s^−1^, where the interface crosses 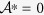. The grey dotted lines denote magnetic field lines. The aspect ratio is equal to one in the image.

**Figure 4:**
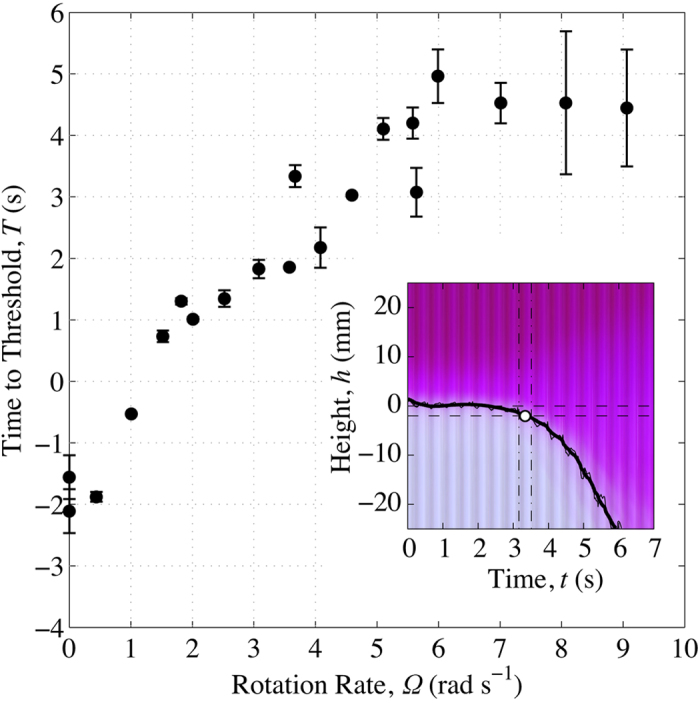
Time *T* elapsed since the descent of the platform halted until the amplitude of the undulations in the interface reached a threshold of 2 mm, plotted as a function of rotation rate Ω. Inset: A ‘time series image’ showing the evolution of the instability over time. *t* = 0 s is defined as the time when the descent of the platform halted. In this image, each column of pixels represents a single video frame; the colour of each pixel within this column is determined from the mean red, green and blue intensities of the corresponding rows of pixels in the video frame (see Supplementary Information for details of this procedure). In this example, the rotation rate was Ω = 3.67 rad s^−1^. The height *h* = 0 mm is defined as the position of the interface immediately before the onset of RTI, indicated by the upper horizontal dashed line. The threshold height −2 mm is indicated by the lower horizontal dashed line. The image was contoured to find the position of the interface (black dotted line) and filtered to remove experimental noise (solid black line). The point at which the interface crosses the threshold height is the ‘time to threshold’ and is indicated by the white circle. Vertical dot-dashed lines indicate the uncertainty in the threshold time *T* (as described in the main text), derived from these fits.

**Figure 5:**
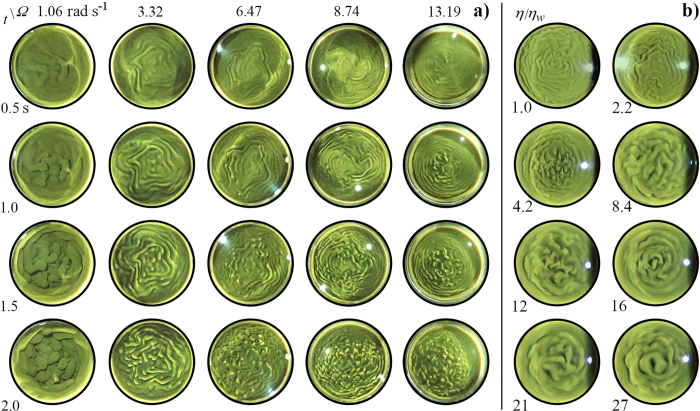
Images of the interface of the Rayleigh-Taylor instability under rotation. **a**) Time increases in increments of 0.5 seconds from top to bottom, rotation rate (rad s^−1^) increases from left to right. An example videos is included in the Supplementary Information, on-line. **b**) Effect of viscosity: dynamic viscosity (in units of the viscosity of water *η*_*w*_ = 1.005 × 10^−3^ kg m^−1^s^−1^ at 22 °C) increases from top left to bottom right. Here, Ω = 7.8 ± 0.1 rad s^−1^.

**Figure 6:**
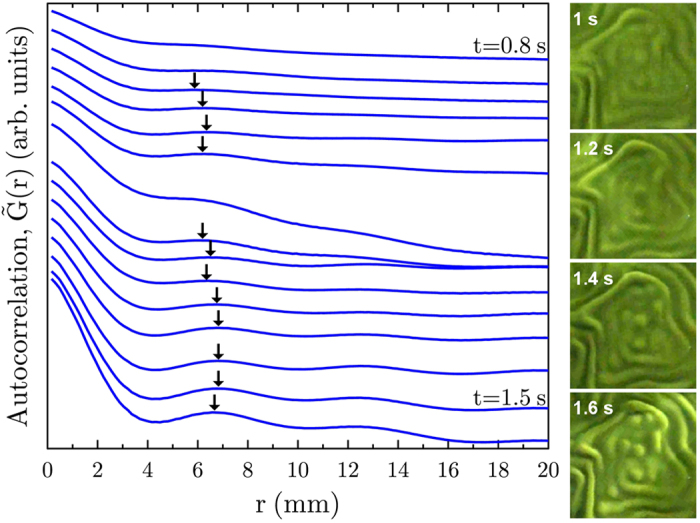
Left: Radially-averaged autocorrelation function 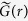; traces offset for clarity. These data are for Ω = 3.3 rad s^−1^; similar data are obtained for other values of Ω. The position of the first peak in each trace is indicated by arrows. Right: Images of the of the interrogation window, *I*, from which the radially-averaged 2D autocorrelation function was calculated, at sequential times after the appearance of the first peak in 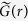.

**Figure 7:**
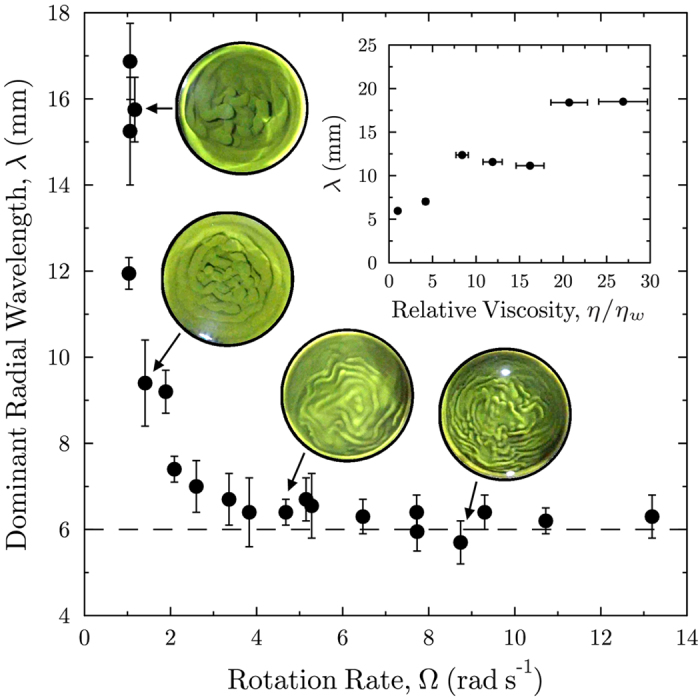
Plot of the dominant initial radial wavelength *λ* as a function of rotation rate. Dashed line: approximate lower wavelength limit observed, *λ* ≈ 6 mm. Inset shows the effect of viscosity (in units of the viscosity of water, *η*_*w*_) on *λ*, for Ω = 7.8 rad s^−1^.

In experiments to study the growth rate of the instability, red water-tracing dye (Cole-Parmer 00295-18) was added to the lighter paramagnetic solution to aid visibility of the growing wave-like interface. The layers had dimensions 250 ml volume and 45 mm radius. We placed a PTFE platform below the descending drive shaft to halt the descent at a position below where the development of RTI plumes became prominent in a non-rotating tank, where the effective Atwood number at the centre of the interface was 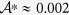. The descent speed was controlled between 8 and 11 mm s^−1^ by the addition of brass weights to the top of the cylindrical tank. We observed no correlation between descent speed and the appearance of RTI fingers. We imaged the interface using a high-speed video camera (Sony NEX-FS100), running at 60 frames per second. To avoid lensing effects caused by the curved cylindrical walls, the cylindrical tank was submerged in a water-filled flat-sided transparent box, as shown in Fig. 2, and filmed through the sides of the box.

In experiments to determine the wavelength of the most rapidly growing mode, red and blue water-tracing dyes (Cole-Parmer 00295-18 & -16) were added to the denser saline solution to make this lower layer opaque. Small quantities of fluorescein sodium salt (~10^−4^ kg l^−1^), were added to this solution to enhance visual contrast between the two layers^25^. The layers had dimensions 300 ml volume, 53.5 mm radius. We imaged the interface from above the liquid tank, with the same high speed camera running at 240 frames per second. Prior to analysis, the high speed video images of the interface were processed, mapping each image into the rotating reference frame of the tank. In the resulting video images, the tank appears stationary.

## Additional Information

**How to cite this article**: Baldwin, K. A. *et al.* The Inhibition of the Rayleigh-Taylor Instability by Rotation. *Sci. Rep.* **5**, 11706; doi: 10.1038/srep11706 (2015).

## Supplementary Material

Supplementary Information

Supplementary Information

Supplementary Information
