# Corrigendum: The Inhibition of the Rayleigh-Taylor Instability by Rotation

**DOI:** 10.1038/srep26574

**Published:** 2016-05-31

**Authors:** Kyle A. Baldwin, Matthew M. Scase, Richard J. A. Hill

Scientific Reports 5: Article number: 1170610.1038/srep11706; published online: 07012015; updated: 05312016

The authors of this Article would like to clarify a point regarding the experimental technique described in the Results section. This clarification does not affect the findings and results of this study.

“Equivalently, we can consider that the force results from a change in the effective density 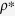 of the liquid in the magnetic field. In this picture the net vertical body force per unit volume is given by *F*_*z*_ = − 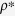
*g*, where 
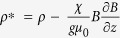
, and the effective Atwood number is then simply defined as 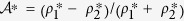, where 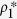 is the effective density of the upper fluid layer, and 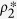 is the effective density of the lower fluid layer. In manipulating the relative magnitudes of the effective densities, the equilibrium profile is unaffected, and the RTI is initiated from hydrostatic conditions”.

should read:

“Equivalently, we can consider that the force results from a change in the effective weight of the liquid in the magnetic field. In this picture the net vertical body force per unit volume is given by *F*_*z*_ = − 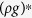, where 
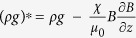
, and the effective Atwood number is then simply defined as 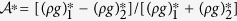, where 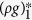 is the effective weight of a unit volume of fluid in the upper layer, and 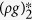 is the effective weight of a unit volume of fluid in the lower layer. In manipulating the relative magnitudes of the effective weights magnetically, the equilibrium profile changes continuously from ‘concave’ to ‘convex’ as it passes through 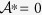, allowing for the instability to be turned on smoothly via the magnetic field, and to develop from approximately hydrostatic conditions”.

