# Supplementary material for: The Inhibition of the Rayleigh-Taylor Instability by Rotation
Source: Sci Rep. 2015 Jul 1;5:11706. doi: 10.1038/srep11706 (PMC4486928; doi:10.1038/srep11706)
Supplement: Supplementary Information [file srep11706-s3.pdf]

The Inhibition of the Rayleigh-Taylor Instability by Rotation: Supplementary Information

Kyle. A. Baldwin, Matthew. M. Scase, and Richard. J. A. Hill

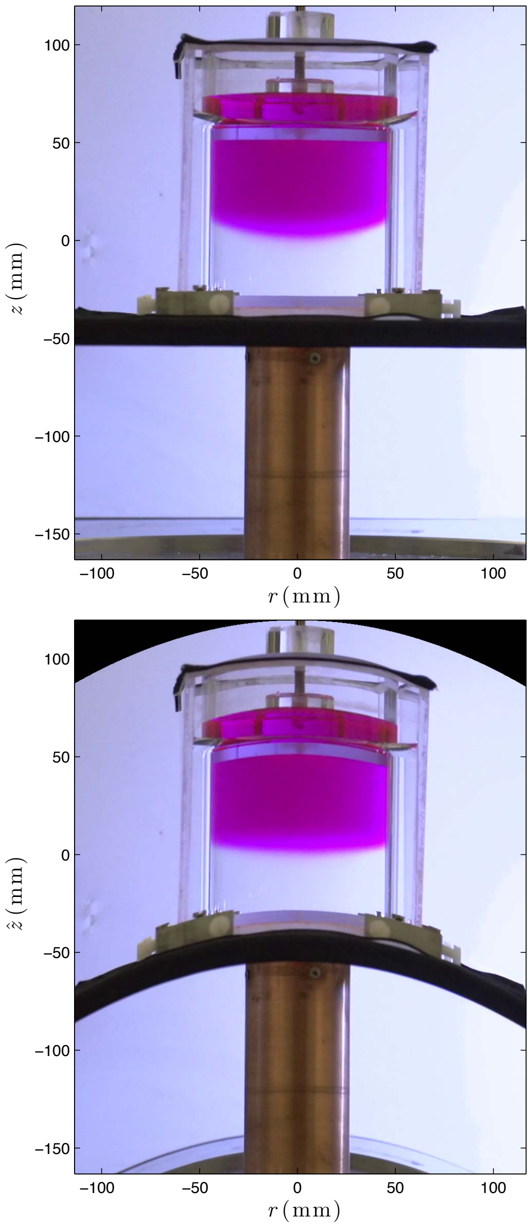

FIG. S1. The mapping of the movie frames to remove the effect of rotation before averaging.

IMAGE PROCESSING: CONSTRUCTION OF TIME SERIES IMAGES

To construct the time series image we employ stretched coordinates to remove the effects of rotation, such that the initial interface is approximately horizontal in the new coordinate system, as opposed to being parabolic in the un-stretched coordinates. A frame from the stretched coordinate movie ($\Omega = 7.01\,\mathrm{rad\,s^{-1}}$) is shown in Fig. S1. The chosen mapping is $r \mapsto r$, $z \mapsto z - \Omega^2 r/2g$. The mean intensity profile is calculated by averaging horizontally in the stretched coordinate system. The mean intensity field is the 'time series' image used to track the mean position of the interface against time, and shown in the inset of Fig. 4 of the main manuscript. The time series is contoured (green field) to extract the position of the interface. Small oscillations in the measured position of the interface are removed using a fifth-order Butterworth filter, the scale of the oscillations is recorded as a measure of the uncertainty in the 'time to threshold'. The time at which the interface crosses the threshold 2 mm below where the interface would have been in the absence of RTI is measured and this is recorded as the time to threshold. The process requires no curve-fitting.

EFFECT OF SALT CONDUCTIVITY

In the experiments involving $NaCl_{(aq)}$ solutions, for each experimental run, the tank was filled with solution drawn from a single, large volume (20 l), in order to ensure that the density and susceptibility of the liquids was the same for each run. For experiments involving $ZnSO_{4(aq)}$ solution (see main text), we attempted to match the density of the $ZnSO_{4(aq)}$ solution to the $NaCl_{(aq)}$ solution. Several solutions of $ZnSO_{4(aq)}$ were prepared with different concentrations of salt. We compared the time taken for the amplitude of the instability to reach a threshold (defined as T in the main text) in a non-rotating experiment, in experiments with NaCl and $ZnSO_4$. We fine tuned the concentration of $ZnSO_4$ until T was the same in both NaCl and $ZnSO_4$ experiments. Since this method actually matches the susceptibility to density ratio χ/ρ of the two liquids there may be a marginal difference in density between the two solutions. Importantly, however, this method matches the *effective* densities of the two liquids in the magnetic field, which is the important parameter in the experiments, rather than the actual density, since it is the effective density that appears in the Atwood number.

Fig. S2 is a set of time-lapse images comparing the growth the RTI between sodium chloride and zinc sulphate solutions of varying concentration, with rotation rates $\Omega = 0$, 2 and 6 rad s^{-1}. These experiments suggests

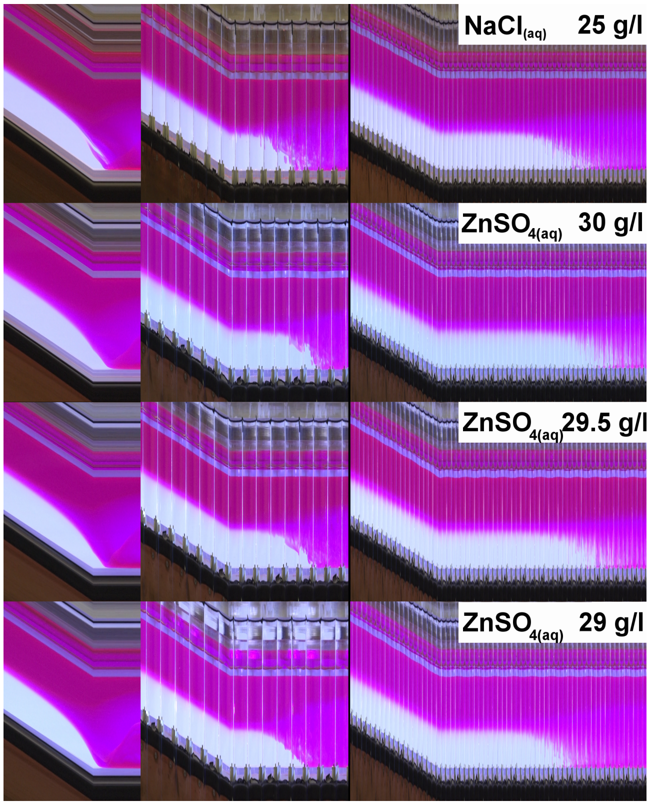

FIG. S2. Comparison between the growth of the RTI for different salt solutions and rotation rates. Rows represent the different salt solutions of the bottom layer used in each experiment (sodium chloride in the topmost, zinc sulphate in the bottom three, with salt concentration labelled in mass of salt added for every litre of water), and columns represent rotation rates $\Omega = 0$, 2 and 6 rad s^{-1}, from left to right. The concentrations, given in g/l in the images, are the masses of NaCl and $ZnSO_4 \cdot 7H_2O$ added to 1 litre of water. This is not equivalent to the densities of the resulting solution, which are approximately equal. (These time-lapse images are formed from the central column of pixels of each video frame, such that the horizontal axis of each image representing elapsed time.)

that the growth rate is most similar for zinc sulphate solutions made by adding 29.5 grams of $ZnSO_4 \cdot 7H_2O$ to 1 litre of water (compare the images in the first and third rows). In these solutions, the difference between the growth of the RTI in non-rotating and rotating experiments is minimal. We conclude that reducing the electrical conductivity, and thus reducing the action of the Lorentz force on the fluid, has minimal effect on the growth rate of the instability under rotation, and the growth rate and character of the instability under rotation is primarily influenced by the restorative effect of the Coriolis force.

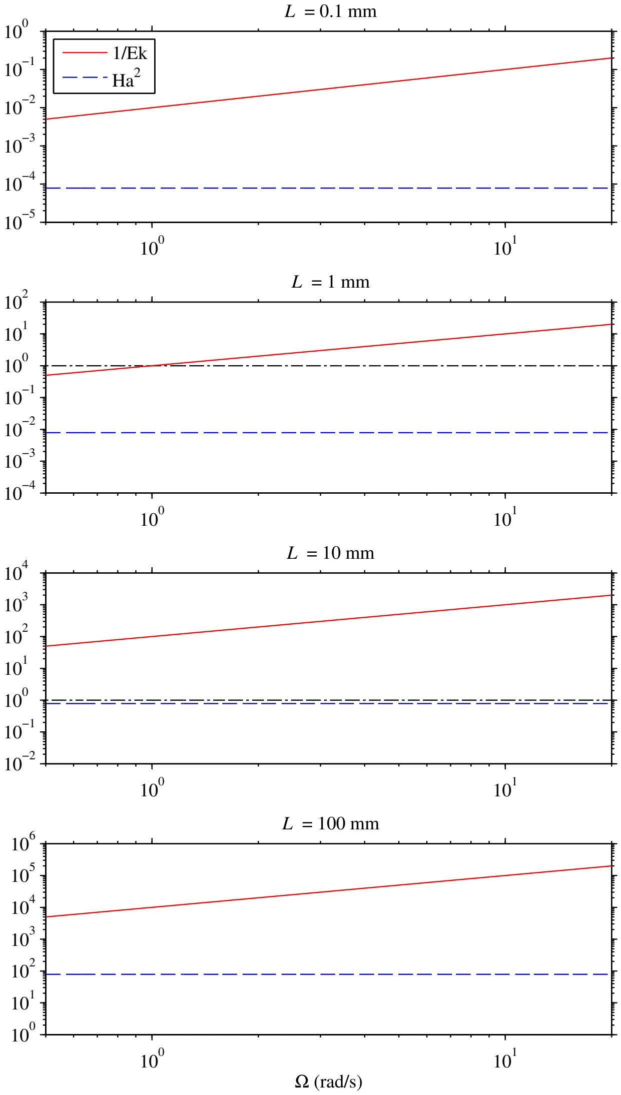

FIG. S3. The unbroken red lines shows how the ratio of Coriolis to viscous forces ($\mathrm{Ek}^{-1} = \Omega L^2/\nu$) varies with rotation rate Ω for various length scales $L = 0.1, 1, 10$ and 100 mm, for a viscosity $\nu = 1 \times 10^{-6} \mathrm{m}^2 \mathrm{s}^{-1}$. The blue long-dashed lines show the ratio of Lorentz to viscous forces ($\mathrm{Ha}^2 = B^2 L^2 \sigma/(\rho\nu)$) for comparison, for $B = 1.4$ T, $\sigma = 4\,\mathrm{S\,m}^{-1}$ and $\rho = 1000\,\mathrm{kg\,m}^{-3}$.

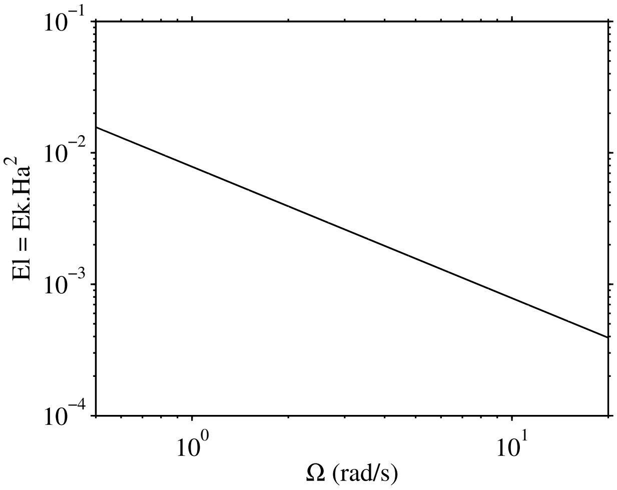

FIG. S4. Dependence of the Elsasser number $\mathrm{El} = \mathrm{Ek.Ha}^2 = \sigma B^2/(\rho\Omega)$, on rotation rate Ω for $B = 1.4$ T, $\sigma = 4\,\mathrm{S\,m^{-1}}$ and $\rho = 1000\,\mathrm{kg\,m^{-3}}$. The Elsasser number is the ratio of Lorentz forces to Coriolis forces.
